# Expansion and adaptive evolution of the *WRKY* transcription factor family in *Avicennia* mangrove trees

**DOI:** 10.1007/s42995-023-00177-y

**Published:** 2023-05-23

**Authors:** Xiao Feng, Guohong Li, Weihong Wu, Haomin Lyu, Jiexin Wang, Cong Liu, Cairong Zhong, Suhua Shi, Ziwen He

**Affiliations:** 1 State Key Laboratory of Biocontrol, Guangdong Key Laboratory of Plant Resources, School of Life Sciences, Southern Marine Science and Engineering Guangdong Laboratory (Zhuhai), Sun Yat-sen University, Guangzhou, 510275 China; 2 Greater Bay Area Institute of Precision Medicine (Guangzhou), Fudan University, Guangzhou, 511458 China; 3 Hainan Academy of Forestry (Hainan Academy of Mangrove), Haikou, 571100 China

**Keywords:** Adaptation, *Avicennia*, Mangrove, Transcription factor, Whole-genome duplication, WRKY

## Abstract

Mangroves are adapted to intertidal zones, which present extreme environmental conditions. WRKYs are among the most prominent transcription factors (TFs) in higher plants and act through various interconnected networks to regulate responses to multiple abiotic stressors. Here, based on omic data, we investigated the landscape and evolutionary patterns of WRKYs in the main mangrove genus *Avicennia*. We found that both the number and the proportion of TFs and WRKYs in *Avicennia* species exceeded their inland relatives, indicating a significant expansion of WRKYs in *Avicennia*. We identified 109 *WRKY* genes in the representative species *Avicennia marina*. Comparative genomic analysis showed that two recent whole-genome duplication (WGD) events played a critical role in the expansion of *WRKY*s, and 88% of *Avicennia marina WRKY*s (*AmWRKY*s) have been retained following these WGDs. Applying comparative transcriptomics on roots under experimental salt gradients, we inferred that there is high divergence in the expression of WGD-retained *AmWRKY*s. Moreover, we found that the expression of 16 *AmWRKY*s was stable between freshwater and moderately saline water but increased when the trees were exposed to high salinity. In particular, 14 duplicates were retained following the two recent WGD events, indicating potential neo- and sub-functionalization. We also found that WRKYs could interact with other upregulated genes involved in signalling pathways and natural antioxidant biosynthesis to enhance salt tolerance, contributing to the adaptation to intertidal zones. Our omic data of the WRKY family in *A. marina* broadens the understanding of how a TF family relates to the adaptive evolution of mangroves.

**Supplementary Information:**

The online version contains supplementary material available at 10.1007/s42995-023-00177-y.

## Introduction

Plant adaptations to extreme environments is a popular research topic in evolutionary biology. Intertidal zones, which represent the interface between land and sea, are associated with a combination of extreme conditions, including high salinity, hypoxia, intense UV light, tidal fluctuations, and high temperature (Giri et al. 2011). Nevertheless, mangrove trees have thrived in such extreme habitats and have evolved specialized phenotypes, such as salt tolerance, aerial roots, and vivipary (Ball 1988; Friess et al. 2019; Tomlinson 2016), indicating that they constitute an ideal model for research on adaptive evolution. Previous studies have investigated mangrove trees from physiological, ecological, and genomic perspectives (Feng et al. 2021; He et al. 2019, 2020; Huang and Wang 2010; Lyu et al. 2018; Ouyang and Lee 2020; Richards et al. 2020; Xu et al. 2017). However, few studies have focused on the evolution of specific gene families, especially those of transcription factors (TFs), due to the insufficiency of high-quality genomic and transcriptomic data.

Gene duplication can lead to the generation of new functional genes and is therefore an important driver of evolution (Innan and Kondrashov 2010; Lynch 2002). Whole-genome duplication (WGD), an important method through which genes can be duplicated, has been widely observed in plants and is believed to contribute to novel environmental adaptations (Clark and Donoghue 2018; Van de Peer et al. 2017, 2021). Plants have evolved a series of strategies to combat unfavourable environmental conditions. Stress signalling pathways are critical in plant responses to external abiotic and biotic stressors (Fujita et al. 2006; Xiong and Zhu 2001; Zhu 2016). These pathways include signal perception, signal transduction, and activation of stress-responsive genes. TFs, which are central to the regulation of gene expression, are involved in these pathways by enhancing or suppressing downstream stress-responsive genes. Usually, a single TF can modulate different kinds of downstream genes. Different TFs can also interact with each other resulting in complex regulatory networks. These abilities allow the robustness of stress-response pathways under complex environmental stress. In plants, approximately 10% of protein-coding genes encode TFs. To date, a wide range of TFs, such as WRKYs, MYBs, bZIPs, NACs, and bHLHs, have been thoroughly documented via functional analysis, such as knockdown/knockout or overexpression experiments, as being involved in the stress response and ultimately contributing to adaptive evolution (Khan et al. 2018). Genetic engineering has been a popular research topic for elucidating the regulatory mechanism and enhancing stress tolerance in plants (Mickelbart et al. 2015; Wang et al. 2003).

WRKYs compose one of the largest TF families in higher plants and are particularly widespread in green plants (Rushton et al. 2010; Ülker and Somssich 2004). Each WRKY TF has at least one WRKY domain within its N-terminal region. This domain is approximately 60 amino acid residues long and contains a conserved WRKYGQK heptapeptide. WRKY TFs also have a zinc-finger motif of either C_2_H_2_ or C_2_HC at their C-terminus. WRKY proteins can be classified into three groups based on their number of WRKY domains and the structure of their zinc-finger motifs. Group I WRKYs contain two sets of WRKY domains and C_2_H_2_ zinc-finger motifs, while the WRKYs of the other groups have only one. Group II WRKYs can be further divided into five subgroups according to their sequence features. Group III WRKYs contain a distinct C_2_HC motif. The first *WRKY* gene, *SPF1*, was cloned from sweet potato in 1994 (Ishiguro and Nakamura 1994). Since then, many *WRKY* genes have been revealed, cloned, and investigated in a variety of plant species, such as *Arabidopsis*, rice, wheat, soybean, and cotton (Jiang et al. 2017; Phukan et al. 2016). Genome-wide identification has also been performed in various plant species (Du et al. 2022; Li et al. 2020; Liu et al. 2020; Nan and Gao 2019; Ross et al. 2007; Song et al. 2018; Wu 2005; Xu et al. 2016a; Zhang et al. 2021). The important biological functions of WRKYs in response to different kinds of abiotic and biotic stressors have been well demonstrated in these studies. Advances in genomic studies have provided higher quality genomes, allowing better identification and characterization of *WRKY*s, which ultimately can help to reveal the origin, diversification, and adaptation of this gene superfamily.

**Fig. 1:**
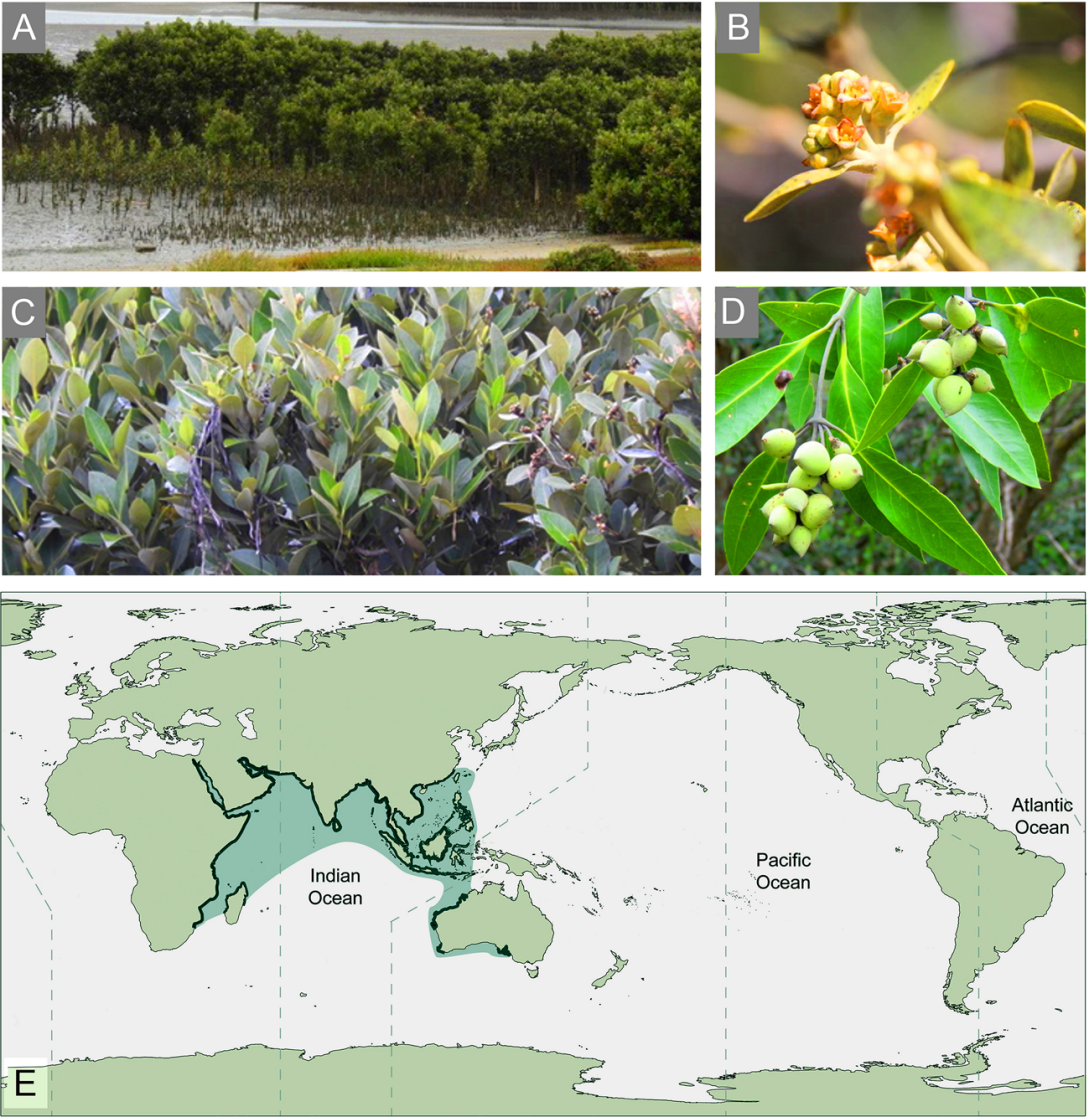
Morphology and distribution of *Avicennia marina*. **A** Bushes. **B** Flowers. **C** Leaves. **D** Fruits (**A**–**D** from Global Biodiversity Information Facility contributed by Graeme Rigg, Stephen Fricker, Graeme Rigg, and Ian Cowan, respectively). **E** The global distribution of *A. marina* is shown in bottle green

*Avicennia marina* is a typical mangrove species and is distributed in the Indo-West Pacific (IWP) region (Fig. 1). Considering its wide distribution range and high-salt tolerance, it is regularly called a “pivot mangrove species”. In addition, this species has evolved a series of adaptive traits, including specialized aerial roots and salt secretion glands (Tomlinson 2016). Previous studies revealed that transposable element load reduction, amino acid usage preference, WGD events, and highly divergent regions are relevant to the adaptation of *Avicennia* (Friis et al. 2021; He et al. 2020; Lyu et al. 2018). However, how specific gene families, especially those of TFs, participate in these adaptations is still poorly understood. In this study, we investigated the landscape and evolutionary pattern of WRKYs in *Avicennia* based on high-quality genomic and transcriptomic data. Using the *WRKY* family as a specific case, we sought to gain new insights into the roles of TFs in the adaptation of mangrove trees to high salinity and improve our understanding of adaptive evolution of plants living in extreme environments.

## Results

### *Avicennia* mangrove trees encode a relatively large number of TFs, especially WRKYs

In a previous study, after combining Illumina short reads, PacBio SMRT long read sequencing data, and Hi-C technology, we obtained a high-quality chromosome-level genome of *A. marina* (Fig. 1) (He et al. 2020). The genome is composed of 32 pseudochromosomes with a total length of 453.2 Mb. Recently, we also de novo assembled high-quality reference genomes of six other mangrove species in the genus *Avicennia* (He et al. 2022). These genomic data are suitable for a comprehensive analysis of the evolution of gene families in the major mangrove genus *Avicennia*.

**Fig. 2:**
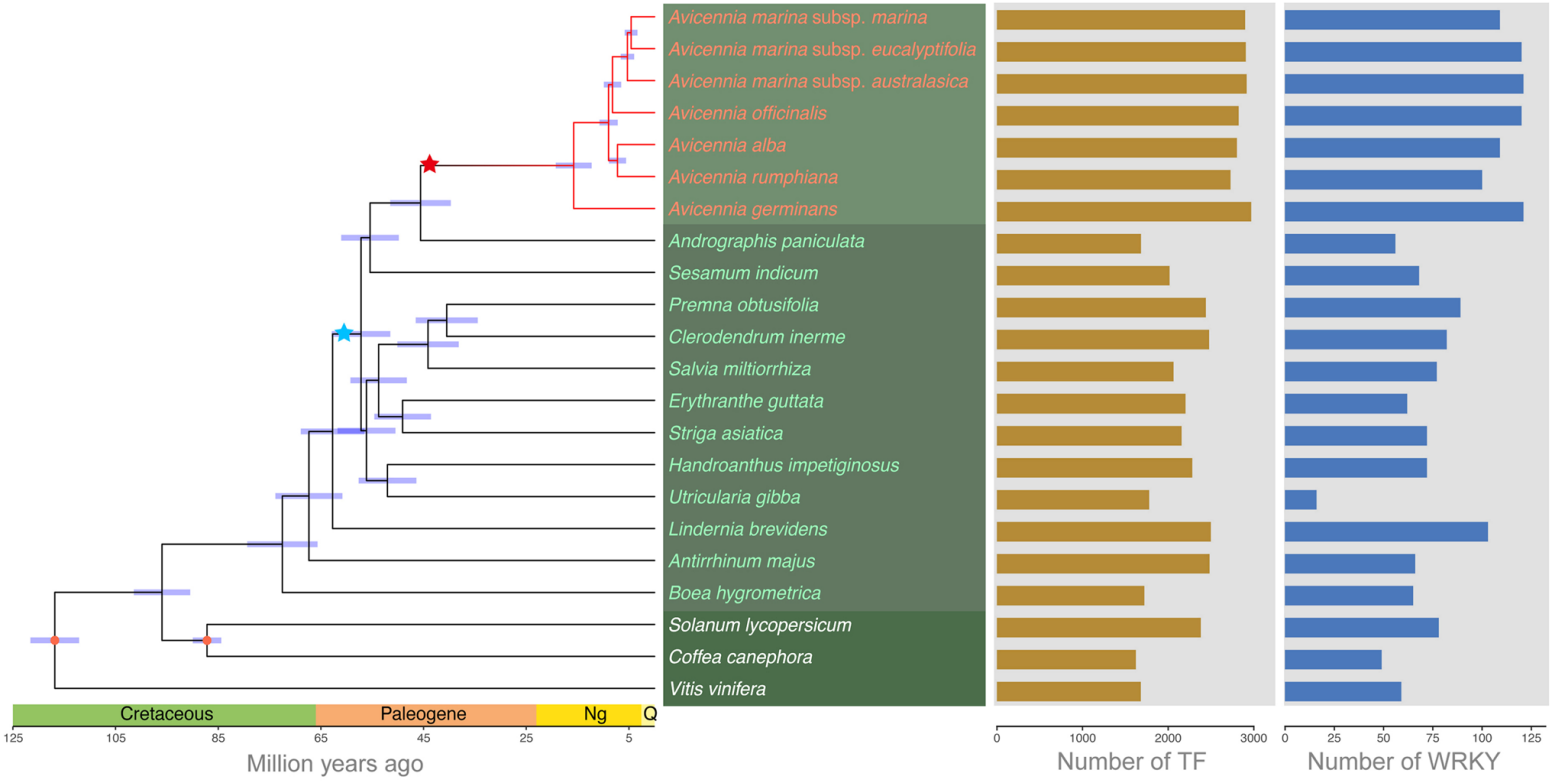
Expansion of TFs and *WRKY* genes in the *Avicennia* lineage. Phylogenetic tree of 22 eudicots, namely, seven *Avicennia* species and subspecies (red), 12 other Lamiales plant species (green), and three outgroups (white). The node bars represent 95% confidence intervals, and the red nodes indicate two calibration nodes. The stars represent the phylogenetic positions of two recent WGD events in *Avicennia*. The gene numbers of all the TFs and *WRKY*s among these plant species are shown on the right

TFs, which are central to the regulation of gene expression, form superfamilies with hundreds to thousands of copies and play important roles in adaptive evolution. The size of TF gene families varies considerably among organisms, correlating with organismal complexity (Levine and Tjian 2003; Shiu et al. 2005). To explore the pattern of TFs in *Avicennia*, we collected 12 other representative plant species in the order Lamiales for which genomic data were available, encompassing 10 families (Acanthaceae, Pedaliaceae, Lamiaceae, Phrymaceae, Orobanchaceae, Bignoniaceae, Lentibulariaceae, Linderniaceae, Plantaginaceae, and Gesneriaceae), as well as *Solanum lycopersicum*, *Coffea canephora*, and *Vitis vinifera* (Supplementary Tables S1, S2), and performed a comparative genomic analysis. Phylogenetic relationships can help infer evolutionary trajectories (Hancock and Edwards 2014). To correctly place *Avicennia* within the order Lamiales, we reconstructed the phylogeny of 22 eudicots, i.e., seven *Avicennia* species and subspecies, 12 other Lamiales species, and three outgroup species. After identifying, aligning, and trimming, we identified 1008 low-copy orthogroups. Using concatenated orthogroup alignments, we constructed a phylogenetic tree by RAxML-NG (Supplementary Fig. S1) and estimated divergence times using MCMCTree with two reliable calibrations. The results were generally consistent with those of the well-known APG IV system (The Angiosperm Phylogeny Group 2016), except that *Handroanthus impetiginosus* was placed in the Bignoniaceae family. *Avicennia* diverged from *Andrographis* approximately 45.60 Mya, while speciation within the genus *Avicennia* mainly occurred was approximately 15.76 Mya (Fig. 2). We identified 2727–2969 TFs encoded in the different *Avicennia* species, accounting for 8.66%-9.56% of the total genes in each species (Fig. 2; Supplementary Fig. S2). The number and proportion of TFs ranked highest in the *Avicennia* species among the 22 plant species and subspecies (*T* test, *P* < 7 × 10^–5^); *Lindernia brevidens* also had a large number of TFs, which may be due to their involvement in the novel desiccation tolerance of this species (Phillips et al. 2008; VanBuren et al. 2018). These results suggested the TFs in *Avicennia* mangroves have undergone expansion, which might contribute to adaptation to extreme environments.

WRKYs, which constitute one of the most prominent transcriptional regulator families in higher plants, are involved in various interconnected regulatory networks in response to multiple abiotic stressors (Phukan et al. 2016). Therefore, we focused on the WRKY TF family to investigate the landscape and evolutionary patterns in *Avicennia*. We initially identified putative *WRKY* genes in each species by integrating TF-specific classification with iTAK and homology-based prediction with PfamScan (Supplementary Table S3). The WRKY domain structure is crucial because of its indispensability for DNA binding and protein complex formation. We strictly filtered ambiguous members lacking the complete WRKYGQK heptapeptide or several variants (such as WRKYGKK, WRKYGEK, WRKYGRK) according to the alignment data. And 100–121 *WRKY* genes were identified among different *Avicennia* species, which accounted for approximately 4% of their total TFs (Supplementary Fig. S2). Using the same workflow (Supplementary Fig. S3), we identified 16–89 *WRKY* genes among 11 other Lamiales plant species as well as 103 in *L. brevidens*, 78 in *S. lycopersicum*, 49 in *C. canephora*, 59 in *Vitis vinifera*, and 72 in the model plant species *Arabidopsis thaliana* (Fig. 2; Supplementary Table S3). We concluded that *WRKY* gene families had significantly expanded in the *Avicennia* lineage compared with other Lamiales species (Supplementary Fig. S2). In addition, the results were consistent with previous reports of genome-wide *WRKY*s in *A. thaliana* (Eulgem et al. 2000), *C. canephora* (Dong et al. 2019), and *V. vinifera* (Wang et al. 2014), although there were slightly fewer than those reported in *S. lycopersicum* (Huang et al. 2012), which indicated that our identification method was accurate and robust. This method further solidified the substantial expansion of *WRKY*s in the *Avicennia* lineage.

### *WRKY* genes are well categorized into (sub)groups

**Fig. 3:**
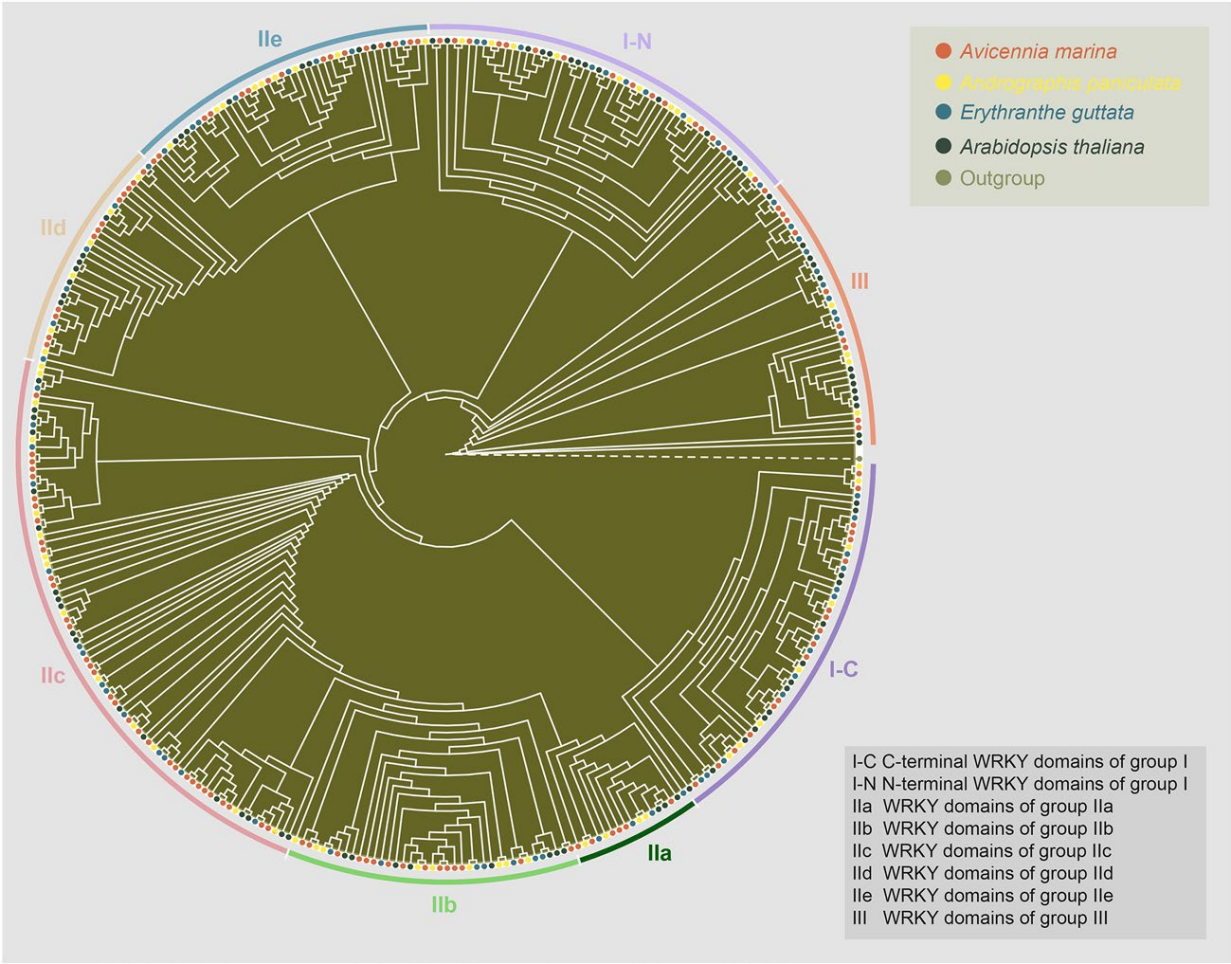
Phylogenetic tree of WRKY domains from *Avicennia marina*, the closely related plant *Andrographis paniculata*, and two model plant species (*Erythranthe guttata* and *Arabidopsis thaliana*). The colours of the nodes represent WRKY domains from these four plant species and an outgroup. The colours of the arcs indicate different groups (or subgroups) of the WRKY domain. I-N and I-C indicate the N-terminal and C-terminal WRKY domains, respectively, of group I

To further categorize and investigate the evolutionary relationship of *WRKY* genes in *Avicennia*, we selected representative plant species and, using RAxML-NG with the Jones‒Taylor‒Thornton (JTT) model, constructed a phylogenetic tree of WRKY domains within 109 *AmWRKY*s in *A. marina*, 56 *ApWRKY*s in the closely related plant species *Andrographis paniculata*, and 62 *EgWRKYs* and 72 *AtWRKYs* in the two model plant species *Erythranthe guttata* and *A. thaliana,* respectively (Kozlov et al. 2019). The WRKY domain sequence from the unicellular protist *Giardia lamblia* was used as the outgroup. The maximum likelihood phylogenetic tree showed that the *WRKY* gene family members could be classified into three major groups and that WRKY domains could be assigned to eight (sub)groups, namely, I-N (N-terminal WRKY domains of group I), I-C (C-terminal WRKY domains of group I), IIa, IIb, IIc, IId, IIe, and III (Fig. 3; Supplementary Table S4). The N-terminal and C-terminal WRKY domain clusters of group I were separated into different clades, indicating the occurrence of parallel evolution. The accuracy of this classification was also verified by the annotations of the best homologues in *A. thaliana* of the *WRKY* genes of the other three species. Interestingly, WRKY domains in the same subgroup of different species were more similar than were other members in the same species. In some subclades, the *WRKY* gene topology was generally in accordance with the species topology (for example, the *Andrographis* genes are sister group members of the *Avicennia* genes). In contrast, the topology in other subclades was more complex, suggesting the occurrence of gene duplication and loss events. Specifically, among the 109 *AmWRKY*s, 17 members belonged to group I, 82 belonged to group II, and 10 belonged to group III. Group II was further divided into five subgroups (IIa-e), which included 6, 15, 33, 12 and 16 members, respectively (Supplementary Figs. S4, S5, S6; Supplementary Table S4). Moreover, *WRKY*s in group II, especially subgroup IIc, were found to have significantly expanded in *A. marina*.

### Genomic location and duplication events among *AmWRKY* genes

WGD events are prevalent throughout the evolutionary history of plants and are speculated to contribute to novel environmental adaptations (Clark and Donoghue 2018; Van de Peer et al. 2017, 2021). WGD events provide an abundance of genetic material with the potential to evolve novel functions, and WGD is an effective way through which many gene families can expand and diversify, especially gene families involved in signal transduction and transcriptional regulation (Blanc and Wolfe 2004). Since the well-known γ-WGD event occurred in core eudicots*, A. marina* has experienced two subsequent WGD events (Xu et al. 2023). Cytogenetically, however, *A. marina* is currently considered a normal diploid organism. Therefore, we were interested in whether preferential retention following a WGD event is an essential method for *AmWRKY* expansion. We first named these *AmWRKY*s located on 28 chromosomes *AmWRKY1* to *AmWRKY 109* (Supplementary Table S5) according to the order of their positions and further identified 97 high-confidence *AmWRKY*s that contain zinc-finger motifs (C_2_H_2_ or C_2_HC). Using BLASTP and MCScanX, we scanned the genome of *A. marina* and identified 396 syntenic block pairs with a minimum of five shared genes. The extensive syntenic block pairs confirmed that WGD events occurred in the recent past. Synonymous substitution rates (Ks) within paralogous gene pairs of syntenic blocks were subsequently calculated. The bimodal mode of the Ks distribution showed two recent WGD events (Supplementary Fig. S7). We found that 96 *AmWRKY*s were retained following the recent WGD events, while 91 members were high-confidence *AmWRKY*s.

**Fig. 4:**
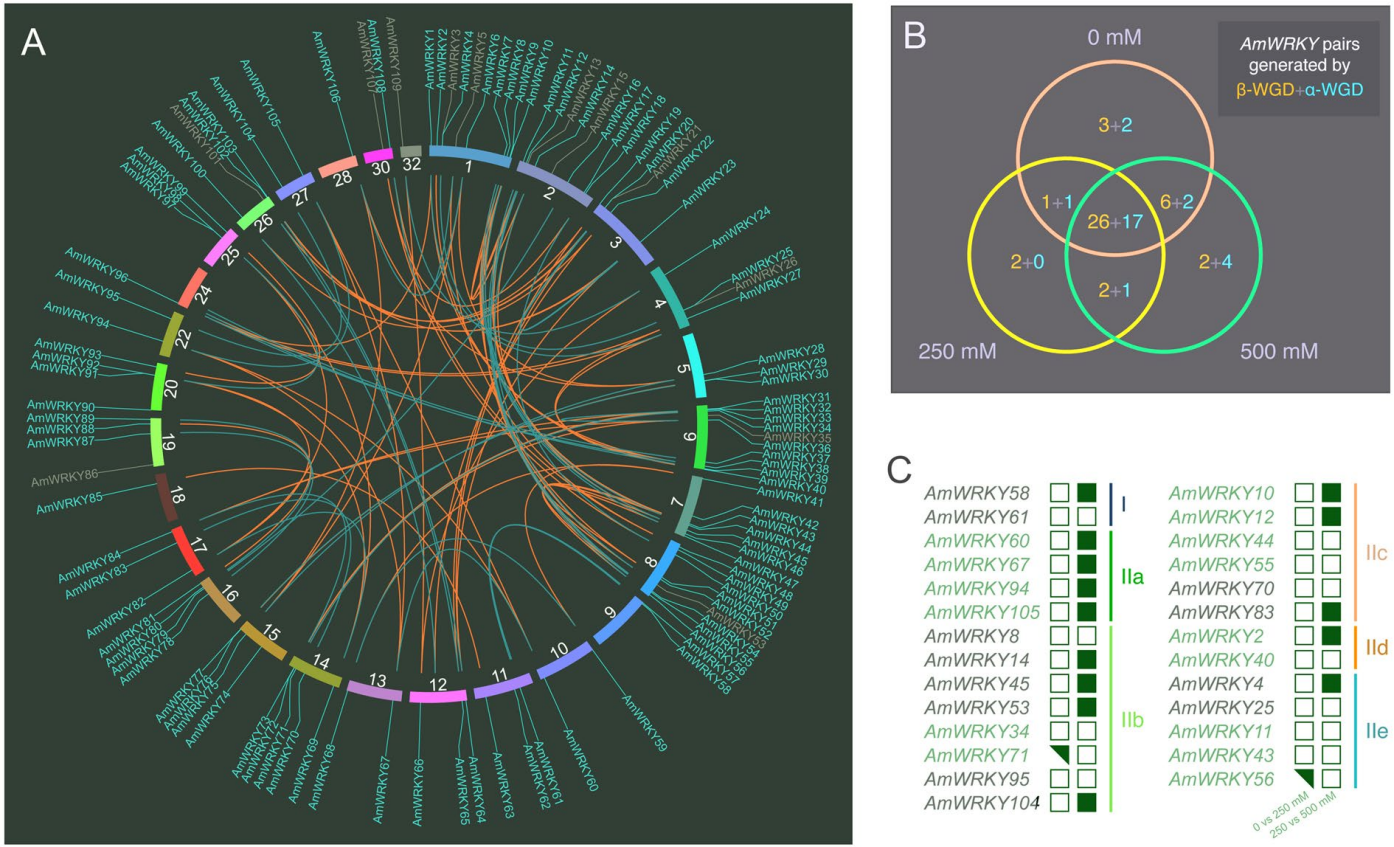
Duplication and expression patterns of *AmWRKY* genes. **A** Chromosomal distribution and relationships of *AmWRKY* genes. Each linking line in the circle centre connects a pair of *AmWRKY* genes generated by two recent WGD events. The blue lines indicate the *Avicennia* lineage-specific WGD (α-WGD) event, while the red lines indicate a slightly earlier recent WGD (β-WGD) event. The circular track presents the 28 chromosomes with *AmWRKY* genes. The approximate distribution of each *AmWRKY* gene is marked on the circular track. The *AmWRKY* gene names with blue colours indicate high-confidence *WRKY* genes. The number on the inner track indicates the chromosome number. **B** Venn diagram of differentially expressed WGD-retained *AmWRKY* pairs among the three salinity conditions. The orange and blue numbers indicate counts of *AmWRKY* pairs generated by β-WGD and α-WGD events, respectively. **C** Expression patterns of WGD-retained *AmWRKY* genes in root tissue across salinity conditions. The solid squares represent upregulation, solid triangles represent downregulation, and hollow squares represent no significant difference in expression. Continuous genes with the same greyscale colour are paralogues generated as a result of the two recent WGD events

Large-scale gene losses after WGD events and different nucleotide substitution rates make it difficult to distinguish duplicate pairs from specific WGD events when only Ks distributions are used. To determine the expansion patterns of *AmWRKY*s resulting from the two most recent WGD events (called α and β), we performed a phylogenetic analysis to reconstruct a species tree and gene trees of each *WRKY* homologous gene group (Supplementary Fig. S8). We integrated all syntenic duplicates related to *WRKY* genes in *A. marina* generated by the two recent WGD events and assigned them to eight four-copy, 11 three-copy, and 19 two-copy groups (see the Materials and methods section). Considering *A. marina* undergoing a lineage-specific α-WGD and sharing a slightly earlier β-WGD event with *E. guttata*, we reconstructed the gene tree of each different-copy group with corresponding orthologues from *E. guttata* and *A. thaliana* to distinguish *AmWRKY*s derived from the two different WGD events (Supplementary Fig. S8). We inferred that duplicates in the four-copy group were generated by the two WGD events and were completely retained, and that the two WGD events also resulted in the generation of duplicates in the three-copy group with a one-copy loss. In the two-copy groups, 13 duplicate pairs were generated by the α-WGD event, while six pairs were generated by the β-WGD event (Fig. 4A). In addition, we found that seven duplicates lost WRKY domains, all of which were generated by the α-WGD event. Integrating Ks-base and phylogenetic approaches, we ultimately identified 33 *AmWRKY* pairs generated by the α-WGD event and 53 *AmWRKY* pairs generated by the β-WGD event. Taken together, these results suggested that the expansion of *WRKY*s in *Avicennia* was a dynamic process and that WGD events played a critical role.

### Expression divergence of two WGD-retained *AmWRKY* genes

Considering that multiple alignments could affect the accuracy of the expression patterns of the *WRKY* gene family members, we first evaluated genetic divergence among the 109 *AmWRKY*s (see Materials and methods). We found that the genetic divergence of these *AmWRKY* gene pairs was greater than 0.158, and the number of mismatches in all the sliding windows (window size = 99 bp) across gene pairs was never less than four base pairs (Supplementary Fig. S9). These results indicated that multiple alignments of RNA sequencing (RNA-seq) reads would not have had any effect on the expression of these *AmWRKY* genes.

The differentiation of retained genes reduces gene redundancy and provides a primary genetic foundation for adaptive evolution. Paralogous *AmWRKY* pairs exhibited greater genetic divergence (Supplementary Fig. S9), indicating sequence differentiation. In addition to sequence divergence, expression divergence is also important. Therefore, we performed an exact conditional test to investigate the expression divergence of retained *AmWRKY* pairs derived from α-WGD and β-WGD events based on transcriptomic data under different salinity conditions (see Materials and methods). As shown in Fig. 4B, we identified 58, 50 and 60 retained *AmWRKY* pairs that were differentially expressed across three different conditions. Moreover, 42 (79.25%) β-WGD-retained *AmWRKY* pairs and 27 (81.82%) α-WGD-retained *AmWRKY* pairs were differentially expressed in at least one condition, while 42 *AmWRKY* pairs were differentially expressed across all three conditions. The Ka/Ks ratios of the *AmWRKY* pairs generated by the α-WGD (mean = 0.339) and β-WGD (mean = 0.293) events were higher than those of all the paralogous genes generated from the two recent WGD events (mean = 0.248). These results showed that most duplicated *AmWRKY*s have evolved under relaxed purifying selection. The results of expression divergence and the Ka/Ks ratio data suggested the potential neo- and sub-functionalization of these *AmWRKY*s under relaxation of purifying selection occurring after the WGD events.

### *AmWRKY* genes involved in high salinity adaptations

High and dynamic salinity hinders plant growth and production (Ball 1998; Singh 2015). Moreover, salinity is the most severe threat to mangrove species in intertidal zones. *WRKY*s act through various regulatory networks to play critical roles in plant responses to biotic and abiotic stressors, secondary metabolite synthesis, and plant development (Jiang et al. 2017; Phukan et al. 2016). To understand the underlying salt tolerance mechanisms induced by WRKYs in *A. marina*, we performed a comparative transcriptome analysis based on salt gradient experimental treatments (see Materials and methods). A 250 mmol/L NaCl concentration is approximately half that of normal seawater NaCl concentration, so we used solutions of 0, 250 and 500 mmol/L NaCl to simulate low, moderate and hypersaline conditions. Using a Tophat2-Cufflinks workflow (Kim et al. 2013; Trapnell et al. 2012), we examined expression profiles and identified differentially expressed genes (DEGs) in the root tissues of plants in two groups (0 compared to 250 mmol/L, and 250 compared to 500 mmol/L; Supplementary Tables S6, S7). We found that 16 *AmWRKY*s were significantly upregulated in the moderate to high-salt comparison group and that two *AmWRKY*s were significantly downregulated in the low-to-moderate salt comparison group (Fig. 4C). Notably, the expression levels of all 16 *AmWRKY*s were stable between low and moderate salinity but increased when the plants were exposed to hyper salinity (Supplementary Fig. S10). Moreover, 14 duplicates of 16 upregulated *AmWRKY*s were retained following the two recent WGD events (Fig. 4C). The compatibility of these genes under moderate salinity indicates that WGD events might shape the regulation of genes induced in response to WRKYs, which might be associated with salt adaptation.

**Fig. 5:**
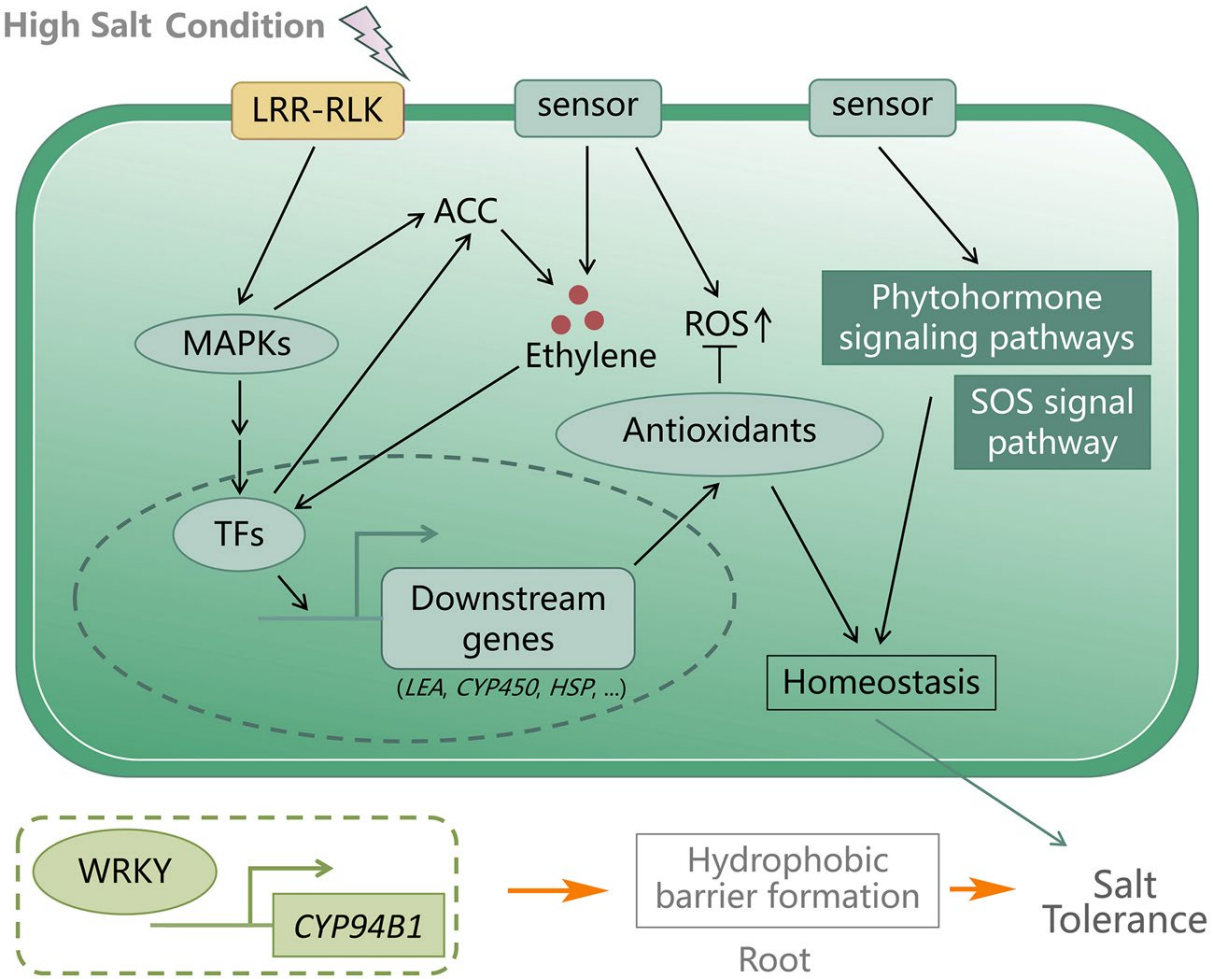
Mechanism of salt tolerance in *A. marina*. The genes upregulated across salinity conditions are involved in the SOS signalling pathway, MAPK signalling pathway, phytohormone signalling pathways, and natural antioxidant biosynthesis. The regulation of root apoplastic barrier formation by WRKY-mediated *CYP94B1* is shown in the lower panel, leading to increased salt tolerance. The content is based on that of Krishnamurthy et al. (2020)

To further explore the regulation induced by WRKYs across salinity conditions, we identified 557 other genes with the same expression pattern as that of the 16 *AmWRKY*s and performed a functional analysis. We found that *AmWRKY*s with this expression pattern were significantly enriched (Fisher’s exact test, *P* < 7.6 × 10^–11^). Several upregulated genes were involved in the salt overly sensitive (SOS) signalling pathway, MAPK signalling pathway, phytohormone (abscisic acid (ABA)-, gibberellin-, jasmonate-, auxin- and ethylene-mediated) signalling pathways, and natural antioxidant biosynthesis (Supplementary Table S8). We also noticed that various genes encoding receptor-like protein kinases, peroxidases, cytochrome P450s, heat shock proteins, and LEA proteins that increased in abundance across salinity conditions might interact with WRKY proteins (Fig. 5). Specifically, the expression of five *LEA* genes (*Am009922, Am009923, Am013278, Am028735,* and *Am029771*) in root tissues increased across salinity conditions. It has been reported that the products of these genes can sequester accumulated ions in cells and act as chaperones to prevent cellular protein aggregation and inactivation to enhance salt tolerance (Battaglia et al. 2008).

## Discussion

This study reports, for the first time, a whole-genome-scale investigation of *WRKY* gene family evolution in mangrove plants, which evolved a series of traits to adapt to intertidal environments. High-quality genomic data provide a rich source of material for investigation and thus lay a reliable foundation for our results.

TFs, which are central to the regulation of gene expression, play a key role in the regulation of plant development and responses to abiotic and biotic stressors. The WRKY family is one of the most prominent TF families in higher plants. Several members of the WRKY family have been shown to be associated with multiple abiotic and biotic stressors (Eulgem et al. 2000; Jiang et al. 2017; Phukan et al. 2016; Rinerson et al. 2015; Rushton et al. 2010, 2012; Ülker and Somssich 2004). We identified the WRKY TFs across the genomes of seven *Avicennia* species and subspecies and 15 non-mangrove plant species for which genomic data were available and found that *WRKY*s had significantly expanded in the *Avicennia* lineage compared with other Lamiales species except *L. brevidens*. Several important TFs involved in dehydration were preferentially retained in *L. brevidens* (VanBuren et al. 2018). Due to tidal fluctuations in the extreme intertidal environment, dynamic and high salinity appears to be the greatest challenge for mangrove trees (Feng et al. 2020). Adaptation to high-salt environments involves a long-term and dynamic process. Salt-responsive genes and important signalling pathways such as the SOS pathway Ca^2+^ signalling pathways, and phytohormone signalling pathways help to enhance plant salt tolerance (Ji et al. 2013; Ryu and Cho 2015; Yang et al. 2019). *WRKY*s also provide tolerance to salt stress, such as *AtWRKY25* and *AtWRKY33* in *A. thaliana*, *DgWRKY1* and *DgWRKY3* in *Dendranthema grandiflorum*, *OsWRKY45* and *OsWRKY72* in rice, *GhWRKY68* in cotton, *TaWRKY10* in *Triticum aestivum*, and *ZmWRKY23* in *Zea mays* (Jiang et al. 2017).

In mangrove trees, WRKYs act through various regulatory networks to play critical roles in adaptation to high-salt concentrations. Taking root tissue as an example, we found that many *AmWRKY*s maintained consistent expression levels between freshwater and moderate-salinity conditions while exhibiting increased expression levels when the plants were exposed to a high-salt environment. Co-expressed genes were involved in the SOS signalling pathway, MAPK signalling pathway, and phytohormone signalling pathways, and various genes involved encode receptor-like protein kinases, cytochrome P450s, heat shock proteins, and LEA proteins. Receptor-like protein kinases are usually involved in the phosphorylation of MAPKs. Thereafter, WRKY proteins can be phosphorylated via MAPK cascades and together regulate ACC synthase activity during ethylene production (Li et al. 2012), which might play a key role in inducing ethylene biosynthesis and signalling in the roots to control growth and development when plants are exposed to high-salt concentrations (Tao et al. 2015). On the other hand, in the endodermis of its roots, *Avicennia* has evolved a hydrophobic barrier that prevents more than 90% of salt from entering the xylem (Krishnamurthy et al. 2014). A transgenic experiment demonstrated that AtWRKY33-mediated *AtCYP94B1* regulates apoplastic barrier formation, leading to increased salt tolerance in *Arabidopsis* (Krishnamurthy et al. 2020). Moreover, secondary oxidation stress is usually caused by hypersalinity stress, resulting in reactive oxygen species (ROS) accumulation. WRKYs regulate downstream genes related to the biosynthesis of natural antioxidants, including glutathione and peroxidase, contributing to scavenging ROS produced in response to high salinity (Anjum et al. 2014). Overall, these *WRKY* genes can maintain cellular environmental homeostasis and can enhance the salt tolerance of mangrove trees, contributing to adaptation to intertidal zones (Fig. 5).

Widely observed WGD events in plants and animals can increase short-term adaptive potential and long-term biological complexity (Dehal and Boore 2005; Van de Peer et al. 2017). WGD doubles the raw genetic material for adaptation, enabling polyploid plants to survive and thrive in extreme environments (He et al. 2020; Van de Peer et al. 2021; Wu et al. 2020). Before the colonization of intertidal zones, several mangrove species independently experienced recent WGD events (Feng et al. 2021; He et al. 2015, 2020; Hu et al. 2020; Xu et al. 2017, 2023). The present study of *AmWRKY*s may provide another perspective of how WGD promoted the adaptive potential of mangrove trees via the expansion of TFs. Since the well-known γ-WGD event occurred in core eudicots*, A. marina* has experienced two subsequent WGD events. We found that 88% of *AmWRKY*s were retained following recent WGD events and inferred that WGD is an essential method through which the *WRKY* gene families expand in *Avicennia* species. The results of our comparative transcriptomic analyses suggested that 14 duplicates of 16 upregulated *AmWRKY*s in mangrove trees under high-salt conditions were retained following the two recent WGD events, pointing towards neo-functionalization. Moreover, based on the transcriptomic data corresponding to each condition, we estimated the expression divergence of retained *AmWRKY* pairs derived from α-WGD and β-WGD events by the exact conditional test. The expression of most of the two WGD-retained *AmWRKY* gene pairs highly diverged, suggesting that there has been potential neo- and sub-functionalization of these *AmWRKY*s under relaxed purifying selection. We also found that seven syntenic duplicates related to *AmWRKY*s had lost their WRKY domain; all of them were generated by the α-WGD event, while five *AmWRKY*s without complete zinc-finger motifs were generated by the α-WGD or β-WGD event. Putative *WRKY* genes that lost their WRKY domain after the β-WGD event may have been deleted due to loss of function. Therefore, the landscape of *WRKY*s in *Avicennia* is the result of a dynamic process driven by gene duplication, relaxed purifying selection and gene loss.

In summary, we investigated the landscape and evolutionary patterns of *WRKYs* in the main mangrove genus *Avicennia* through a combination of genomic and transcriptomic data. We deduced that duplication, expansion, neo- and sub-functionalization of *WRKY*s in mangrove trees could aid in maintaining cellular environmental homeostasis, contributing to adaptation to intertidal environments. This information provides new insights into the roles of TFs in the salt adaptation of mangrove trees and improves our understanding of adaptive evolution of plants living in extreme environments.

## Materials and methods

### Genomic data collection

We obtained whole-genome sequence data and annotation data of seven *Avicennia* mangrove species and subspecies (He et al. 2020, 2022), 12 other Lamiales plant species, i.e., *A. paniculata* (Sun et al. 2019), *Sesamum indicum* (Wang et al. 2015), *Premna obtusifolia*, *Clerodendrum inerme*, *Salvia miltiorrhiza* (Xu et al. 2016b), *E. guttata* (Hellsten et al. 2013), *Striga asiatica* (Yoshida et al. 2019), *H. impetiginosus* (Silva-Junior et al. 2018), *Utricularia gibba* (Lan et al. 2017), *L. brevidens* (VanBuren et al. 2018), *Antirrhinum majus* (Li et al. 2019), and *Boea hygrometrica* (Xiao et al. 2015), *S. lycopersicum* (The Tomato Genome Consortium 2012), *Coffea canephora* (Denoeud et al. 2014), and *V. vinifera* (The French–Italian Public Consortium for Grapevine Genome Characterization 2007). The seven *Avicennia* species and subspecies were *A. marina* subsp. *marina*, *A. marina* subsp. *eucalyptifolia*, *A. marina* subsp. *australasica*, *Avicennia officinalis*, *Avicennia alba*, *Avicennia rumphiana*, and *Avicennia germinans*. Their genomic sequences were generated in our laboratory and are accessible from the National Genomics Data Center (NGDC) or through personal communication. The genomic sequences of the 15 non-mangrove plant species, covering the major families of the Lamiales for which genomic data were available, were downloaded from several genomic databases or obtained via personal communication (Supplementary Table S1). The completeness of the predicted genes was evaluated by Benchmarking Universal Single-Copy Orthologs (BUSCO) v.3.1.0 with the eudicotyledons_odb10 database (Seppey et al. 2019).

### Phylogeny reconstruction and molecular dating

We used OrthoFinder v2.4.0 (Emms and Kelly 2019) to classify orthologous groups from the 22 plant species and subspecies. In addition, we conducted a reciprocal BLASTP best-hit method between the proteins of the 22 species and the single-copy gene set of the BUSCO dataset. Combining the two results above, we identified 1034 low-copy orthogroups. Within each orthogroup, we obtained amino acid alignment via MAFFT v7.429 (Katoh and Standley 2013), converted the data to codon alignments via PAL2NAL v14 (Suyama et al. 2006), trimmed the alignments via Gblocks v0.91b (Castresana 2000) and discarded alignments shorter than 150 bp, after which 1008 orthogroups were retained. Based on the alignments, a phylogenetic tree was constructed, which was inferred via RAxML-NG v0.9.0 with the GTR + GAMMA + I model, with 1000 bootstrap replicates (Kozlov et al. 2019), and with *V. vinifera* included as an outgroup. We further dated the tree via MCMCTree from the PAML v4.9j package with approximate likelihood calculations (Reis and Yang 2011; Yang 2007) based on the calibration times for divergence between *S. lycopersicum* and *C. canephora* (83–89 Mya) and between *C. canephora* and *V. vinifera* (114–125 Mya) (Guyot et al. 2012). After a burn-in consisting of 1,000,000 iterations, 10 million generations of the MCMC process were run and sampled every 500 generations. The MCMC process was performed twice independently to ensure convergence. The R package ggtree was used to visualize the phylogenetic tree (Yu et al. 2017).

### Identification and comparison analysis of WRKY TFs in the order Lamiales

We identified TFs among the 22 high-quality genomic datasets via iTAK v17a and then assigned them to detailed TF families according to their annotations (Zheng et al. 2016). We also performed functional annotations via PfamScan with the Protein family database (Pfam) identifier of the WRKY domain (PF03106) (Finn et al. 2010). Merging these results together, we identified putative genes with WRKY domains. Using only the WRKY domain of each sequence, we then aligned all the putative WRKY proteins of the same species with MAFFT v7.429 (L-INS-i model) (Katoh and Standley 2013). Subsequently, we manually filtered putative proteins without complete WRKYGQK heptapeptide or several variants (WRKYGKK, WRKYGEK, WRKYGRK) to eliminate ambiguous sequences. We also eliminated splice variants and retained only the longest variant for further analysis. Based on the workflow (Supplementary Fig. S3), we successfully identified all *WRKY* gene families among these 22 plant species and subspecies and considered those genes whose products have with a zinc-finger motif as high-confidence *WRKY* genes. We identified the WRKY TF family of the model plant species *A. thaliana* from The Arabidopsis Information Resource (TAIR) database (https://www.arabidopsis.org/browse/genefamily/WRKY.jsp). Almost all of the *AtWRKY*s were high-confidence *WRKY* genes, with one exception: the protein encoded by *AtWRKY19* (*AT4G12020*) did not contain a zinc-finger motif. For *AtWRKY52*, due to the improvement of the genome annotation, *AT5G45270* was omitted, and *AT5G45260* was used to replace it in the TAIR10 genome assembly.

### Multiple sequence alignment, phylogenetic analysis, and classification of *WRKY*s in *A. marina* and its relatives

Using MAFFT v7.429 with the L-INS-i model, we first aligned the WRKY domain sequences of all the identified WRKY proteins in *A. marina*, the closely related plant species *A. paniculata*, two model plant species (*E. guttata* and *A. thaliana*), and the unicellular protist *G. lamblia* (accession EAA40901) as the outgroup (Katoh and Standley 2013). We then constructed a phylogenetic tree inferred via RAxML-NG v0.9.0 (Kozlov et al. 2019) with the JTT model selected by ProtTest v3.4.2 (Darriba et al. 2011; Guindon and Gascuel 2003). Based on the phylogenetic tree and known classification of *AtWRKY*s, we assigned all identified *WRKY*s to different groups and subgroups. To further ensure the accuracy of the classification, we also searched for *A. thaliana* homologues of each *WRKY* in the other three species and retained the best hits.

### Features of *AmWRKY*s

We used a consistent naming pattern for all the *WRKY* genes in *A. marina* for further organization. Each gene name starts with an abbreviation for the species name *A. marina* (*Am*), followed by the TF family name (*WRKY*) and order related to its position in the genome (e.g., *AmWRKY1*, *AmWRKY2*, …). We then generated and visualized the exon‒intron structures of the *AmWRKY* genes via Gene Structure Display Server (GSDS) v2.0 (http://gsds.gao-lab.org) based on their respective gene sequences and positions (Hu et al. 2015).

### Expansion pattern of *AmWRKY*s

WGD is the primary source of duplicate genes in plants, especially genes that encode TFs. To determine the degree of collinearity, we utilized BLASTP to align the protein sequences in *A. marina* with optimal parameters (identity ≥ 30%, e-value < 10^–10^, alignment length ≥ 30% of both query and reference sequence length). Using MCScanX, we identified syntenic blocks with at least five paralogous gene pairs (Wang et al. 2012). Then, we aligned each gene pair and applied KaKs_Calculator v2.0 to calculate Ks with the YN substitution model (Wang et al. 2010). We subsequently visualized the Ks distribution of these gene pairs within *A. marina*. The bimodal Ks distribution showed two rounds of recent WGD events (Supplementary Fig. S7). To identify duplicates from the two recent WGD events in *A. marina*, we calculated the Ks values of all the gene pairs within syntenic blocks and obtained the median Ks of each block. Then, we selected gene pairs in the syntenic blocks whose median Ks was between 0 and 1.2 and filtered the gene pairs with Ks values larger than 1.55. When only Ks distribution information is available, the large-scale gene losses after WGD events make it hard to distinguish from which WGD events these duplicates are derived. Therefore, we applied a phylogenetic approach to detect the WGD pattern among the *AmWRKY*s. We integrated all the paralogous duplicates in *A. marina* generated by the two recent WGD events via the R package igraph (https://igraph.org). Within each *A. marina*, *E. guttata* and *A. thaliana* orthogroup, the orthologues of *AmWRKYs* in the other two species were defined according to the best BLAST hit, and a phylogenetic tree was reconstructed by RAxML-NG v0.9.0 (Kozlov et al. 2019). Because *A. marina* has experienced a lineage-specific WGD event and shares another WGD event with *E. guttata*, we characterized the WGD pattern of *AmWRKY*s according to the phylogenetic tree data and identified different WGD-retained genes among the *AmWRKY*s (Supplementary Fig. S8). Then, we calculated the Ka/Ks ratios of *AmWRKY* pairs derived from the two WGD events using KaKs_Calculator v2.0 with the YN substitution model based on the above alignment results (Wang et al. 2010) and compared them with those of all the paralogous genes generated as a result of the two recent WGD events.

### Transcriptome sequencing and analysis

To understand how salt tolerance is induced by WRKYs in *A. marina*, we performed a comparative transcriptome analysis of root tissues of *A. marina* plants under experimental salt gradient treatments. We obtained RNA-seq data (accession numbers SRR16279087, SRR16279088, SRR16279089, SRR16279090, SRR16279092, and SRR16279093 UNDER BioProject ID PRJNA719266 in the NCBI Sequence Read Archive database) from our previous study (Xu et al. 2023). The experimental details are described by Xu et al. (2023). Three groups of seedlings were irrigated with 0, 250, or 500 mmol/L NaCl solutions for seven days, simulating low, moderate and hypersaline conditions, respectively. Each group contained two biological replicates. Reads of low quality were removed following the protocol described in our previous study (Feng et al. 2020).

We first evaluated the genetic divergence among the 109 *AmWRKY*s. The amino acid alignment of each AmWRKY pair was obtained by MAFFT v7.429 (Katoh and Standley 2013) and then converted to codon alignment data by PAL2NAL v14 (Suyama et al. 2006). Then, using the Kimura two-parameter method, we calculated the genetic divergence of the gene pairs among the 109 *AmWRKY*s (Kimura 1980). We also used a sliding window approach (window length with 99 bp adjusted to the RNA-seq read length) to estimate mismatches of each pair.

The clean RNA-seq reads were then mapped back to the high-quality chromosome-level genome of *A. marina* (He et al. 2020) by TopHat v2.1.1 (Kim et al. 2013) and Bowtie2 v2.2.9 (Langmead and Salzberg 2012) with the option “–read-mismatches 2” to avoid multiple alignments in genome locations belonging to different *AmWRKY*s. The Cufflinks package v2.2.1 (Trapnell et al. 2012) was employed to assemble transcripts and quantify expression. We then identified DEGs in the 0 mmol/L and 250 mmol/L NaCl, and in the 250 mmol/L and 500 mmol/L NaCl, comparison groups. Genes whose expression fold-change was greater than two and whose q-value was less than 5% were considered differentially expressed. *AmWRKY* expression patterns were characterized, with expression increasing with salt concentration “upregulated”, a change in the opposite direction “downregulated”, and “no significant difference” for the remaining genes. To further explore the regulation of the *AmWRKY*s across salinity conditions, we identified key co-expressed genes in *A. marina* (defined as BLASTP hits against the content of the *A. thaliana* TAIR10 genome, with a cut-off e-value < 10^−10^).

We also investigated the expression divergence of retained *AmWRKY* pairs derived from the α-WGD and β-WGD events in the three conditions. The number of reads uniquely mapped to each gene for each condition was determined using HTSeq (v1.99.2) (Anders et al. 2015). We then performed an exact conditional test to determine the differential expression of each pair of duplicate *AmWRKY*s (Gu et al. 2008), which has been applied at length in both soybean and *Brassica* (Liu et al. 2014; Roulin et al. 2013). The *P* value was computed from the exact conditional test using the R function *binom.test()* for each pair of duplicate *AmWRKY*s. Multiple testing was corrected by applying the Bonferroni correction method. We considered gene pairs with a corrected *P* value below 5% differentially expressed. Only gene pairs whose read number was greater than 0 were included in the analysis. We ultimately identified WGD-retained *AmWRKY* pairs with differential expression for each condition based on the consistency between two biological replicates.

## Supplementary Information

Below is the link to the electronic supplementary material.

Supplementary file1 (DOCX 8670 KB)

## Data Availability

All the data generated and analysed during this study are included in this published article and its supplementary files.
