# Supplementary material for: Expansion and adaptive evolution of the WRKY transcription factor family in Avicennia mangrove trees
Source: Mar Life Sci Technol. 2023 May 23;5(2):155–68. doi: 10.1007/s42995-023-00177-y (PMC10232687; doi:10.1007/s42995-023-00177-y)
Supplement: Supplementary file 1 — Supplementary file1 (DOCX 8670 KB) [file 42995_2023_177_MOESM1_ESM.docx]

**Supplementary Information**

**Expansion and adaptive evolution of the *WRKY* transcription factor family in *Avicennia* mangrove trees**

Xiao Feng^1,2^, Guohong Li^1^, Weihong Wu^1^, Haomin Lyu^1^, Jiexin Wang^1^, Cong Liu^1^, Cairong Zhong^3^, Suhua Shi^1,*^, Ziwen He^1,*^

^1^ State Key Laboratory of Biocontrol, Guangdong Key Laboratory of Plant Resources, School of Life Sciences, Southern Marine Science and Engineering Guangdong Laboratory (Zhuhai), Sun Yat-sen University, Guangzhou 510275, China

^2^ Greater Bay Area Institute of Precision Medicine (Guangzhou), Fudan University, Guangzhou 511458, China

^3^ Hainan Academy of Forestry (Hainan Academy of Mangrove), Haikou 571100, China

* Correspondence should be addressed to Ziwen He (heziwen@mail.sysu.edu.cn) and Suhua Shi (lssssh@mail.sysu.edu.cn).

**Fig. S1.** **Maximum likelihood tree of 22 eudicots, including seven *Avicennia* species and subspecies, 12 other Lamiales plants, and three outgroup taxa**. Numbers at the nodes indicate bootstrap support value, and 1000 bootstraps were used.


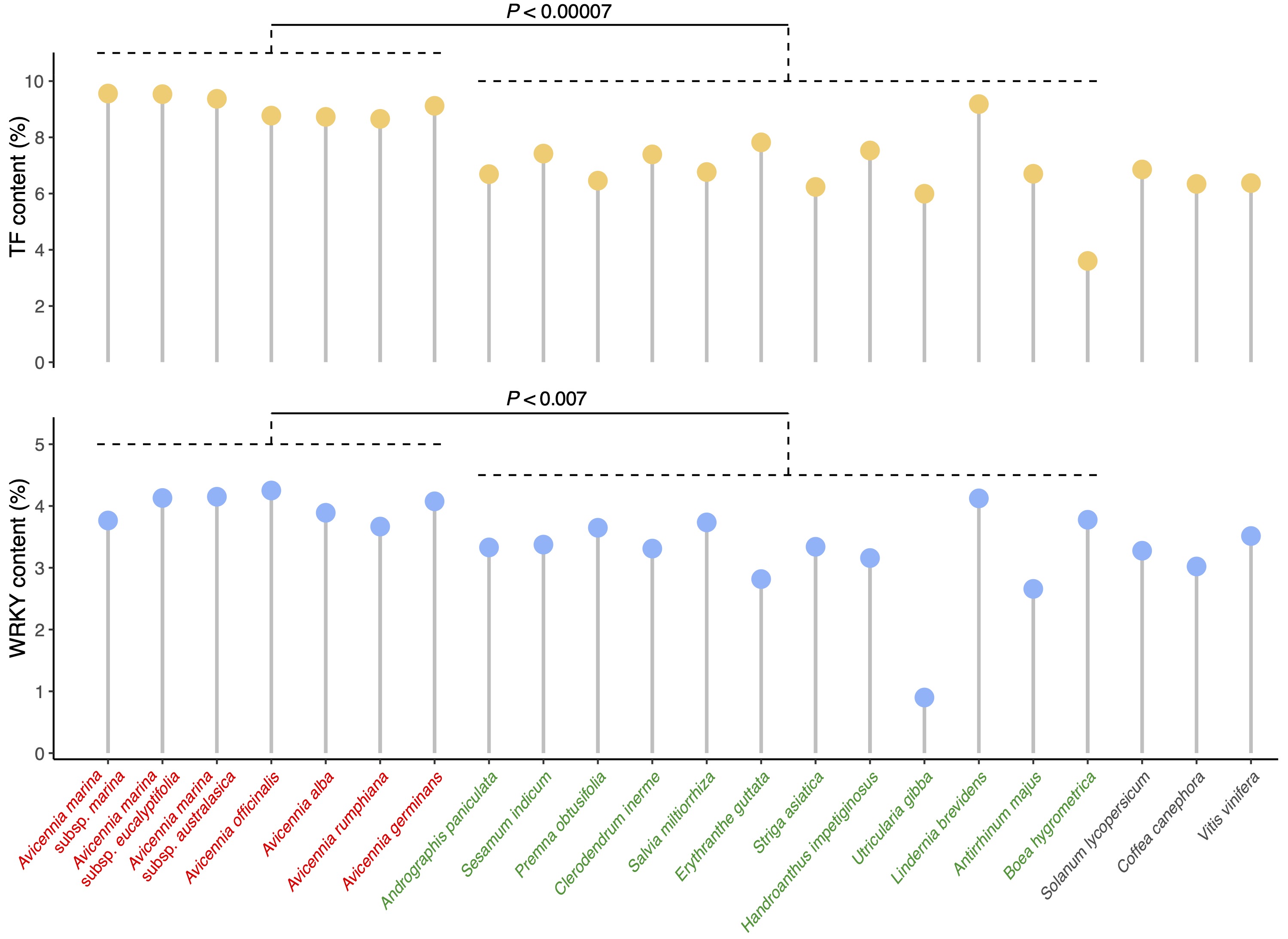


**Fig. S2.** **The proportions of transcription factors (TFs) and the proportions of WRKYs in TFs among 22 eudicots, including seven *Avicennia* species and subspecies (red), 12 other Lamiales plants (green), and three outgroup taxa (gray).**

**Fig. S3. Overview of workflow for identification of transcription factors and *WRKY* family.**

**Fig. S4. Phylogenetic tree of WRKY domains from *Avicennia marina*.** A thousand bootstraps were used and nodes with <50% bootstrap support were collapsed. I-N or I-C means the N-terminal or C-terminal WRKY domains of group I.


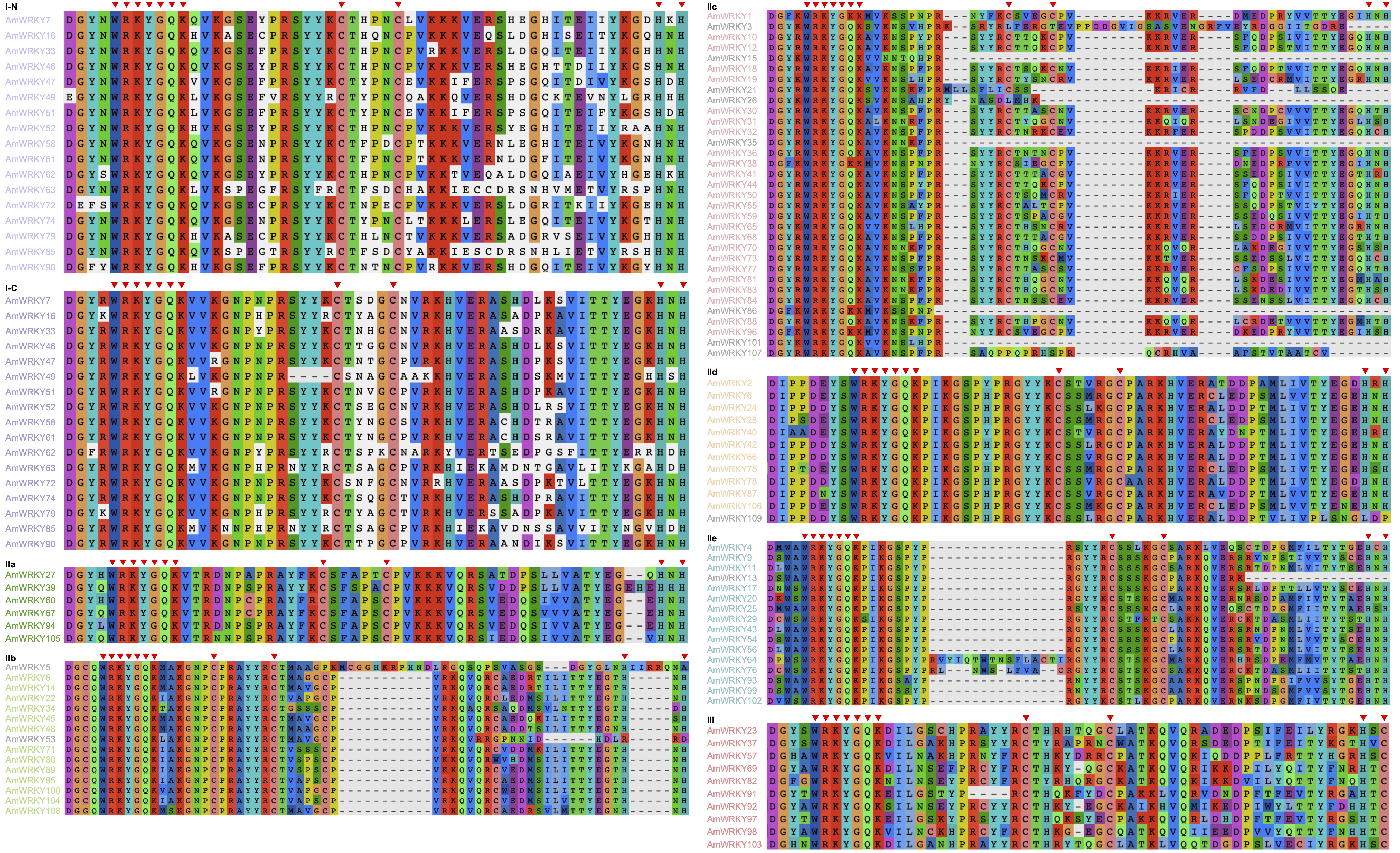


**Fig. S5. Alignment of all AmWRKY domain amino acid sequences.** I-N or I-C means the N-terminal or C-terminal WRKY domains of group I. WRKYGQK heptapeptides and zinc finger motifs are indicated by arrowheads. The AmWRKY protein names with colors except for gray mean high-confidence WRKY genes with complete zinc finger motifs.

**Fig. S6. Exon-intron structures of *AmWRKY* genes.** The structure is generated and displayed by GSDS. The colored boxes and black lines indicate exons and introns, respectively.

**Fig. S7. Ks Distribution of syntenic paralogous genes in *A. marina*.** The bimodal mode shows two rounds of recent WGD events.

**Fig. S8. The expansion pattern inference of *AmWRKY*s by the two recent WGDs.** **A**: Species tree. **B-F**: Gene trees of *WRKY* homologous gene groups. Ama: *Avicennia marina*, Egu: *Erythranthe guttata*, Ath: *Arabidopsis thaliana*.

**
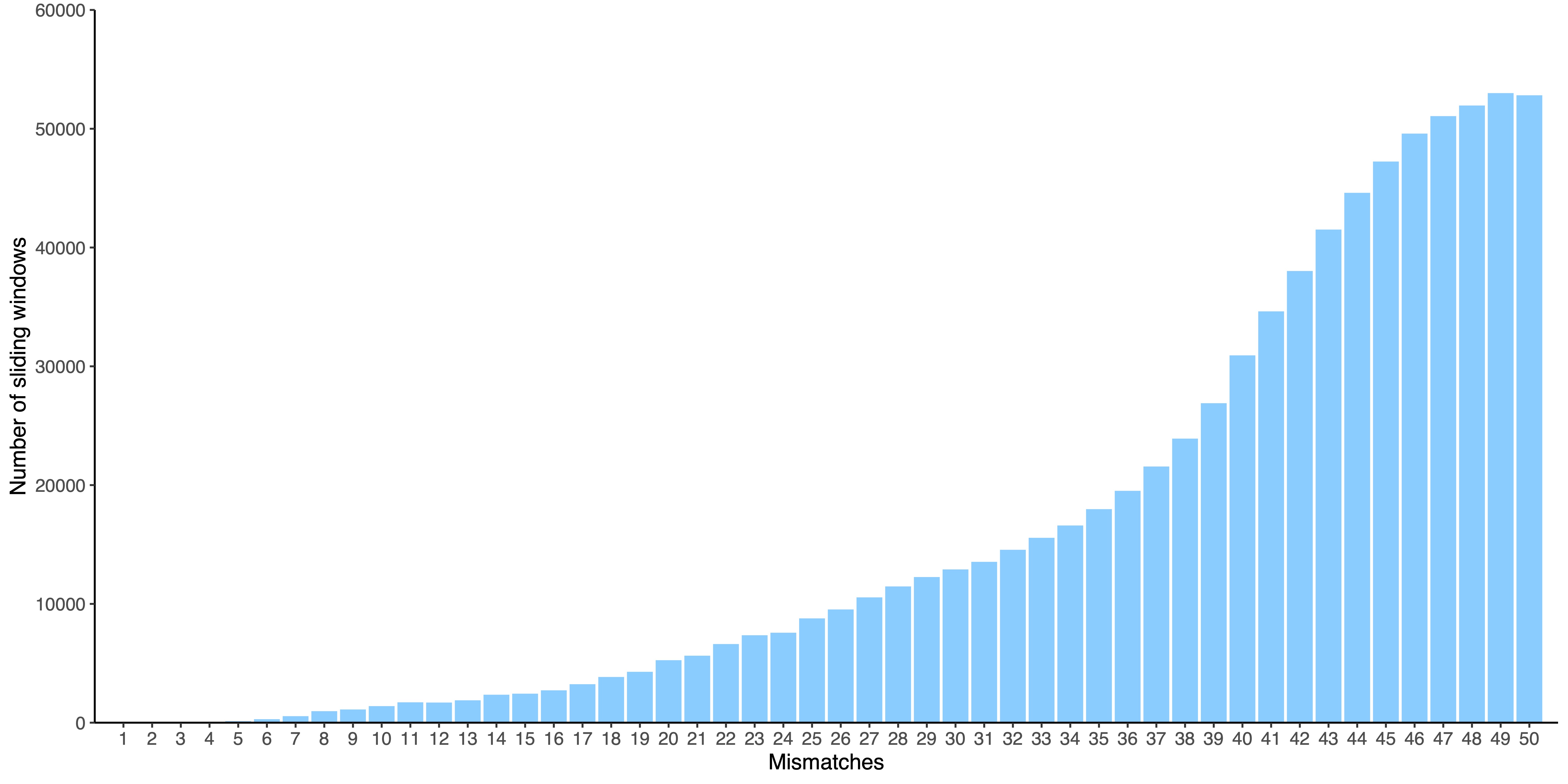
**

**Fig. S9. The distribution of mismatches in sliding windows of each pair among 109 *AmWRKY*s.** The window length is 99 bp, adjusted to RNA-seq read length. The numbers of sliding windows with mismatches more than 50 bp are not shown.

**Fig. S10. Expression heatmap of *AmWRKY* genes in root tissue across salinity contrasts (0, 250, and 500 mM NaCl).** *AmWRKY* genes could be classified into group I, group II (including five subgroups IIa-e) and group III based on the phylogenetic relationship. The scale on the bottom left represents the FPKM value with z-score normalization.

**Table S1. A summary of whole-genome sequences used in this study.**

| Order | Family | Organism | Source | Accession number | Reference |
| --- | --- | --- | --- | --- | --- |
| Lamiales | Acanthaceae | *Avicennia marina* subsp. *marina* | NGDC | GWHBCKT00000000 | He *et al.*, 2020 |
| Lamiales | Acanthaceae | *Avicennia marina* subsp. *eucalyptifolia* | NGDC | GWHBCJE00000000 | He *et al.*, 2022 |
| Lamiales | Acanthaceae | *Avicennia marina* subsp. *australasica* | NGDC | GWHBCJD00000000 | He *et al.*, 2022 |
| Lamiales | Acanthaceae | *Avicennia officinalis* | NGDC | GWHBCJF00000000 | He *et al.*, 2022 |
| Lamiales | Acanthaceae | *Avicennia alba* | NGDC | GWHBCJG00000000 | He *et al.*, 2022 |
| Lamiales | Acanthaceae | *Avicennia rumphiana* | NGDC | GWHBCJH00000000 | He *et al.*, 2022 |
| Lamiales | Acanthaceae | *Avicennia germinans* | NGDC | GWHBCJI00000000 | He *et al.*, 2022 |
| Lamiales | Acanthaceae | *Andrographis paniculata* | NCBI | GCA_004354405.1 | Sun *et al.*, 2019 |
| Lamiales | Pedaliaceae | *Sesamum indicum* | Sinbase |  | Wang *et al.*, 2015 |
| Lamiales | Lamiaceae | *Clerodendrum inerme* | NGDC | GWHBCJK00000000 | He *et al.*, 2022 |
| Lamiales | Lamiaceae | *Premna obtusifolia* | NGDC | GWHBCJJ00000000 | He *et al.*, 2022 |
| Lamiales | Lamiaceae | *Salvia miltiorrhiza* | NDCTCM | ftp://danshen.ndctcm.org:10402/ | Xu *et al.*, 2016 |
| Lamiales | Phrymaceae | *Erythranthe guttata* | Phytozome v12 | https://phytozome.jgi.doe.gov/pz/portal.html#!info?alias=Org_Mguttatus | Hellsten *et al.*, 2013 |
| Lamiales | Orobanchaceae | *Striga asiatica* | Dryad | https://doi.org/10.5061/dryad.53t3574 | Yoshida *et al.*, 2019 |
| Lamiales | Bignoniaceae | *Handroanthus impetiginosus* | GigaDB | http://gigadb.org/dataset/100379 | Silva-Junior *et al.*, 2017 |
| Lamiales | Lentibulariaceae | *Utricularia gibba* | CoGe | https://genomevolution.org/coge/GenomeInfo.pl?gid=29027 | Lan *et al.*, 2017 |
| Lamiales | Linderniaceae | *Lindernia brevidens* | Google Drive | L_brevV1.8 | VanBuren *et al.*, 2018 |
| Lamiales | Plantaginaceae | *Antirrhinum majus* | Snapdragon Genome Database | http://bioinfo.sibs.ac.cn/Am/download_v3.php | Li *et al.*, 2019 |
| Lamiales | Gesneriaceae | *Boea hygrometrica* | NCBI | GCA_001598015.1 | Xiao *et al.*, 2015 |
| Solanales | Solanaceae | *Solanum lycopersicum* | Phytozome v12 | https://phytozome.jgi.doe.gov/pz/portal.html#!info?alias=Org_Slycopersicum | The Tomato Genome Consortium, 2012 |
| Gentianales | Rubiaceae | *Coffea canephora* | Coffee Genome Hub | https://coffee-genome.org/download | Denoeud *et al.*, 2014 |
| Vitales | Vitaceae | *Vitis vinifera* | Phytozome v12 | https://phytozome.jgi.doe.gov/pz/portal.html#!info?alias=Org_Vvinifera | The French–Italian Public Consortium for Grapevine Genome Characterization, 2007 |

NGDC stands for National Genomics Data Center, NCBI stands for National Center for Biotechnology Information, NDCTCM stands for National Data Center of Traditional Chinese Medicine.

**Table S2. BUSCO evaluation of genome annotation completeness.**

| Organism | Complete BUSCOs | Complete and single-copy BUSCOs | Complete and duplicated BUSCOs | Fragmented BUSCOs | Missing BUSCOs |
| --- | --- | --- | --- | --- | --- |
| *Avicennia marina* subsp. *marina* | 1962 (92.5%) | 1786 (84.2%) | 176 (8.3%) | 77 (3.6%) | 82 (3.9%) |
| *Avicennia marina* subsp. *eucalyptifolia* | 1915 (90.3%) | 1720 (81.1%) | 195 (9.2%) | 112 (5.3%) | 94 (4.4%) |
| *Avicennia marina* subsp. *australasica* | 1884 (88.8%) | 1697 (80.0%) | 187 (8.8%) | 123 (5.8%) | 114 (5.4%) |
| *Avicennia officinalis* | 1962 (92.6%) | 1787 (84.3%) | 175 (8.3%) | 87 (4.1%) | 72 (3.3%) |
| *Avicennia alba* | 1981 (93.4%) | 1819 (85.8%) | 162 (7.6%) | 67 (3.2%) | 73 (3.4%) |
| *Avicennia rumphiana* | 1965 (92.7%) | 1809 (85.3%) | 156 (7.4%) | 60 (2.8%) | 96 (4.5%) |
| *Avicennia germinans* | 1881 (88.7%) | 1690 (79.7%) | 191 (9.0%) | 111 (5.2%) | 129 (6.1%) |
| *Andrographis paniculata* | 1585 (74.7%) | 1489 (70.2%) | 96 (4.5%) | 242 (11.4%) | 294 (13.9%) |
| *Sesamum indicum* | 1814 (85.5%) | 1688 (79.6%) | 126 (5.9%) | 108 (5.1%) | 199 (9.4%) |
| *Premna obtusifolia* | 1933 (91.1%) | 1396 (65.8%) | 537 (25.3%) | 58 (2.7%) | 130 (6.2%) |
| *Clerodendrum inerme* | 1844 (86.9%) | 1686 (79.5%) | 158 (7.4%) | 147 (6.9%) | 130 (6.2%) |
| *Salvia miltiorrhiza* | 1733 (81.7%) | 1585 (74.7%) | 148 (7.0%) | 170 (8.0%) | 218 (10.3%) |
| *Erythranthe guttata* | 2062 (97.2%) | 1966 (92.7%) | 96 (4.5%) | 37 (1.7%) | 22 (1.1%) |
| *Striga asiatica* | 1903 (89.7%) | 1738 (81.9%) | 165 (7.8%) | 90 (4.2%) | 128 (6.1%) |
| *Handroanthus impetiginosus* | 1718 (81.0%) | 1453 (68.5%) | 265 (12.5%) | 158 (7.4%) | 245 (11.6%) |
| *Utricularia gibba* | 1533 (72.3%) | 1444 (68.1%) | 89 (4.2%) | 197 (9.3%) | 391 (18.4%) |
| *Lindernia brevidens* | 1865 (87.9%) | 1708 (80.5%) | 157 (7.4%) | 79 (3.7%) | 177 (8.4%) |
| *Antirrhinum majus* | 2012 (94.8%) | 1916 (90.3%) | 96 (4.5%) | 55 (2.6%) | 54 (2.6%) |
| *Boea hygrometrica* | 1667 (78.6%) | 1599 (75.4%) | 68 (3.2%) | 142 (6.7%) | 312 (14.7%) |
| *Solanum lycopersicum* | 2041 (96.3%) | 2025 (95.5%) | 16 (0.8%) | 64 (3.0%) | 16 (0.7%) |
| *Coffea canephora* | 2006 (94.6%) | 1957 (92.3%) | 49 (2.3%) | 64 (3.0%) | 51 (2.4%) |
| *Vitis vinifera* | 1939 (91.4%) | 1895 (89.3%) | 44 (2.1%) | 127 (6.0%) | 55 (2.6%) |

BUSCO stands for Benchmarking Universal Single-Copy Orthologs.

**Table S3. Summary of *WRKY* genes among 22 plants.**

| Organism | Number of Putative *WRKY*s | Number of *WRKY*s |
| --- | --- | --- |
| *Avicennia marina* subsp. *marina* | 116 | 109 |
| *Avicennia marina* subsp. *eucalyptifolia* | 129 | 120 |
| *Avicennia marina* subsp. *australasica* | 125 | 121 |
| *Avicennia officinalis* | 124 | 120 |
| *Avicennia alba* | 114 | 109 |
| *Avicennia rumphiana* | 107 | 100 |
| *Avicennia germinans* | 122 | 121 |
| *Andrographis paniculata* | 60 | 56 |
| *Sesamum indicum* | 71 | 68 |
| *Premna obtusifolia* | 93 | 89 |
| *Clerodendrum inerme* | 87 | 82 |
| *Salvia miltiorrhiza* | 77 | 77 |
| *Erythranthe guttata* | 65 | 62 |
| *Striga asiatica* | 79 | 72 |
| *Handroanthus impetiginosus* | 74 | 72 |
| *Utricularia gibba* | 17 | 16 |
| *Lindernia brevidens* | 106 | 103 |
| *Antirrhinum majus* | 69 | 66 |
| *Boea hygrometrica* | 65 | 65 |
| *Solanum lycopersicum* | 81 | 78 |
| *Coffea canephora* | 49 | 49 |
| *Vitis vinifera* | 59 | 59 |

**Table S4. The numbers of *WRKY* genes in different (sub)groups.**

| Organism | I | IIa | IIb | IIc | IId | IIe | III | Total |
| --- | --- | --- | --- | --- | --- | --- | --- | --- |
| *Avicennia marina* | 17 | 6 | 15 | 33 | 12 | 16 | 10 | 109 |
| *Andrographis paniculata* | 10 | 4 | 9 | 14 | 6 | 9 | 4 | 56 |
| *Erythranthe guttata* | 13 | 4 | 7 | 14 | 6 | 8 | 10 | 62 |
| *Arabidopsis thaliana* | 14 | 3 | 8 | 18 | 7 | 9 | 13 | 72 |

**Table S5. The number of *AmWRKY* genes on chromosomes in different (sub)groups** **in *Avicennia marina*.**

| Chromosome | I | IIa | IIb | IIc | IId | IIe | III | Total |
| --- | --- | --- | --- | --- | --- | --- | --- | --- |
| Chr1 | 1 | 0 | 2 | 3 | 2 | 2 | 0 | 10 |
| Chr2 | 1 | 0 | 1 | 3 | 0 | 3 | 0 | 8 |
| Chr3 | 0 | 0 | 1 | 2 | 0 | 1 | 1 | 5 |
| Chr4 | 0 | 1 | 0 | 1 | 1 | 1 | 0 | 4 |
| Chr5 | 0 | 0 | 0 | 1 | 1 | 1 | 0 | 3 |
| Chr6 | 1 | 1 | 1 | 5 | 1 | 0 | 1 | 10 |
| Chr7 | 2 | 0 | 1 | 2 | 1 | 1 | 0 | 7 |
| Chr8 | 3 | 0 | 2 | 2 | 0 | 2 | 0 | 9 |
| Chr9 | 1 | 0 | 0 | 0 | 0 | 0 | 1 | 2 |
| Chr10 | 0 | 1 | 0 | 1 | 0 | 0 | 0 | 2 |
| Chr11 | 3 | 0 | 0 | 0 | 0 | 0 | 0 | 3 |
| Chr12 | 0 | 0 | 0 | 1 | 1 | 1 | 0 | 3 |
| Chr13 | 0 | 1 | 0 | 1 | 0 | 0 | 0 | 2 |
| Chr14 | 1 | 0 | 1 | 2 | 0 | 0 | 1 | 5 |
| Chr15 | 1 | 0 | 0 | 1 | 1 | 1 | 0 | 4 |
| Chr16 | 1 | 0 | 1 | 1 | 1 | 0 | 0 | 4 |
| Chr17 | 0 | 0 | 0 | 2 | 0 | 0 | 1 | 3 |
| Chr18 | 1 | 0 | 0 | 0 | 0 | 0 | 0 | 1 |
| Chr19 | 0 | 0 | 1 | 2 | 1 | 0 | 0 | 4 |
| Chr20 | 1 | 0 | 0 | 0 | 0 | 1 | 2 | 4 |
| Chr22 | 0 | 1 | 1 | 0 | 0 | 0 | 0 | 2 |
| Chr24 | 0 | 0 | 0 | 1 | 0 | 0 | 0 | 1 |
| Chr25 | 0 | 0 | 0 | 0 | 0 | 1 | 2 | 3 |
| Chr26 | 0 | 0 | 1 | 1 | 0 | 1 | 1 | 4 |
| Chr27 | 0 | 1 | 1 | 0 | 0 | 0 | 0 | 2 |
| Chr28 | 0 | 0 | 0 | 0 | 1 | 0 | 0 | 1 |
| Chr30 | 0 | 0 | 1 | 1 | 0 | 0 | 0 | 2 |
| Chr32 | 0 | 0 | 0 | 0 | 1 | 0 | 0 | 1 |
| Total | 17 | 6 | 15 | 33 | 12 | 16 | 10 | 109 |

**Table S6. The number of differentially expressed genes in root tissues in *Avicennia marina*.**

| Condition | Up-regulated genes | Down-regulated genes |
| --- | --- | --- |
| 0 mM *vs.* 250 mM | 155 | 170 |
| 250 mM *vs.* 500 mM | 607 | 140 |

**Table S7. The expression pattern in root tissues in *Avicennia marina*.**

|  | Up-regulated  (250 mM *vs.* 500 mM) | Down-regulated  (250 mM *vs.* 500 mM) | Non-significant  (250 mM *vs.* 500 mM) |
| --- | --- | --- | --- |
| Up-regulated  (0 mM *vs.* 250 mM) | 4 | 43 | 108 |
| Down-regulated  (0 mM *vs.* 250 mM) | 30 | 1 | 139 |
| Non-significant  (0 mM *vs.* 250 mM) | 573 | 96 | 31971 |

**Table S8. Detailed annotation of up-regulated genes under high salinity.**

| *Avicennia marina* | *Arabidopsis thaliana* | Gene name | Gene description | Function |
| --- | --- | --- | --- | --- |
| *Am002547* | *AT4G21410* | *CRK29* | cysteine-rich RLK (receptor-like protein kinase) 29 | receptor |
| *Am002548* | *AT4G05200* | *CRK25* | cysteine-rich RLK (receptor-like protein kinase) 25 | receptor |
| *Am006216* | *AT4G05200* | *CRK25* | cysteine-rich RLK (receptor-like protein kinase) 25 | receptor |
| *Am009724* | *AT4G21410* | *CRK29* | cysteine-rich RLK (receptor-like protein kinase) 29 | receptor |
| *Am012411* | *AT1G65800* | *RK2* | receptor kinase 2 | receptor |
| *Am012433* | *AT4G21380* | *RK3* | receptor kinase 3 | receptor |
| *Am032087* | *AT5G60900* | *RLK1* | receptor-like protein kinase 1 | receptor |
| *Am033141* | *AT3G22060* |  | receptor-like protein kinase-related family protein | receptor |
| *Am003244* | *AT4G36950* | *MAPKKK21* | mitogen-activated protein kinase kinase kinase 21 | MAPK signaling pathway |
| *Am005744* | *AT5G66850* | *MAPKKK5* | mitogen-activated protein kinase kinase kinase 5 | MAPK signaling pathway |
| *Am007783* | *AT3G45640* | *MPK3* | mitogen-activated protein kinase 3 | MAPK signaling pathway |
| *Am020353* | *AT3G45640* | *MPK3* | mitogen-activated protein kinase 3 | MAPK signaling pathway |
| *Am000410* | *AT3G61510* | *ACS1* | ACC synthase 1 | ethylene signaling pathway |
| *Am007021* | *AT3G61510* | *ACS1* | ACC synthase 1 | ethylene signaling pathway |
| *Am011358* | *AT1G05010* | *EFE* | ethylene-forming enzyme | ethylene signaling pathway |
| *Am020768* | *AT3G23240* | *ERF1* | ethylene response factor 1 | ethylene signaling pathway |
| *Am025357* | *AT3G23240* | *ERF1* | ethylene response factor 1 | ethylene signaling pathway |
| *Am008202* | *AT3G23240* | *ERF1* | ethylene response factor 1 | ethylene signaling pathway |
| *Am006562* | *AT5G50080* | *ERF110* | ethylene response factor 110 | ethylene signaling pathway |
| *Am001607* | *AT5G54510* | *DFL1* | auxin-responsive GH3 family protein | auxin signaling pathway |
| *Am003526* | *AT5G54510* | *DFL1* | auxin-responsive GH3 family protein | auxin signaling pathway |
| *Am006085* | *AT1G20925* | *PILS1* | auxin efflux carrier family protein | auxin signaling pathway |
| *Am008768* | *AT2G14960* | *GH3.1* | auxin-responsive GH3 family protein | auxin signaling pathway |
| *Am020139* | *AT3G25290* |  | auxin-responsive family protein | auxin signaling pathway |
| *Am030716* | *AT2G46370* | *JAR1* | auxin-responsive GH3 family protein | auxin signaling pathway |
| *Am019177* | *AT3G55970* | *JRG21* | jasmonate-regulated gene 21 | jasmonate signaling pathway |
| *Am019526* | *AT5G13220* | *JAZ10* | jasmonate-zim-domain protein 10 | jasmonate signaling pathway |
| *Am022783* | *AT5G13220* | *JAZ10* | jasmonate-zim-domain protein 10 | jasmonate signaling pathway |
| *Am026295* | *AT1G19180* | *JAZ1* | jasmonate-zim-domain protein 1 | jasmonate signaling pathway |
| *Am012696* | *AT1G30040* | *GA2OX2* | gibberellin 2-oxidase | gibberellin signaling pathway |
| *Am032987* | *AT1G78440* | *GA2OX1* | gibberellin 2-beta-dioxygenase | gibberellin signaling pathway |
| *Am005708* | *AT1G45249* | *ABF2* | abscisic acid responsive elements-binding factor 2 | ABA signaling pathway |
| *Am001294* | *AT2G30360* | *SIP4* | SOS3-interacting protein 4 | SOS signaling pathway |
| *Am006637* | *AT1G21550* | *F24J8_15* | calcium-binding EF-hand family protein | calcium signaling pathway |
| *Am007420* | *AT2G26190* | *T1D16_17* | calmodulin-binding family protein | calcium signaling pathway |
| *Am010375* | *AT3G22930* | *CML11* | calmodulin-like 11 | calcium signaling pathway |
| *Am012613* | *AT4G18700* | *CIPK12* | CBL-interacting protein kinase 12 | calcium signaling pathway |
| *Am013509* | *AT5G57010* | *MHM17_13* | calmodulin-binding family protein | calcium signaling pathway |
| *Am013964* | *AT3G10300* |  | calcium-binding EF-hand family protein | calcium signaling pathway |
| *Am016887* | *AT4G25800* | *F14M19_80* | calmodulin-binding protein | calcium signaling pathway |
| *Am025053* | *AT5G39670* | *MIJ24_17* | calcium-binding EF-hand family protein | calcium signaling pathway |
| *Am026640* | *AT2G41010* | *CAMBP25* | calmodulin (CAM)-binding protein of 25 kDa | calcium signaling pathway |
| *Am030011* | *AT4G38810* | *T9A14_90* | calcium-binding EF-hand family protein | calcium signaling pathway |
| *Am033562* | *AT3G17510* | *CIPK1* | CBL-interacting protein kinase 1 | calcium signaling pathway |
| *Am008341* | *AT1G02930* | *GSTF6* | glutathione S-transferase 6 | ROS scavenging |
| *Am012746* | *AT3G09270* | *GSTU8* | glutathione S-transferase TAU 8 | ROS scavenging |
| *Am013069* | *AT5G02790* | *GSTL3* | glutathione S-transferase family protein | ROS scavenging |
| *Am018622* | *AT2G29420* | *GSTU7* | glutathione S-transferase tau 7 | ROS scavenging |
| *Am004446* | *AT2G41480* | *PRX25* | peroxidase superfamily protein | ROS scavenging |
| *Am011604* | *AT3G01190* | *T4P13_12* | peroxidase superfamily protein | ROS scavenging |
| *Am014176* | *AT3G03670* | *T12J13_5* | peroxidase superfamily protein | ROS scavenging |
| *Am029519* | *AT5G05340* | *PRX52* | peroxidase superfamily protein | ROS scavenging |
| *Am000296* | *AT5G45340* | *CYP707A3* | cytochrome P450, family 707, subfamily A, polypeptide 3 | related to salt tolerance |
| *Am000545* | *AT4G12320* | *CYP706A6* | cytochrome P450, family 706, subfamily A, polypeptide 6 | related to salt tolerance |
| *Am007166* | *AT5G45340* | *CYP707A3* | cytochrome P450, family 707, subfamily A, polypeptide 3 | related to salt tolerance |
| *Am012785* | *AT5G36110* | *CYP716A1* | cytochrome P450, family 716, subfamily A, polypeptide 1 | related to salt tolerance |
| *Am013175* | *AT3G52970* | *CYP76G1* | cytochrome P450, family 76, subfamily G, polypeptide 1 | related to salt tolerance |
| *Am015049* | *AT3G48520* | *CYP94B3* | cytochrome P450, family 94, subfamily B, polypeptide 3 | related to salt tolerance |
| *Am015504* | *AT5G36110* | *CYP716A1* | cytochrome P450, family 716, subfamily A, polypeptide 1 | related to salt tolerance |
| *Am018384* | *AT3G48270* | *CYP71A26* | cytochrome P450, family 71, subfamily A, polypeptide 26 | related to salt tolerance |
| *Am020510* | *AT5G24910* | *CYP714A1* | cytochrome P450, family 714, subfamily A, polypeptide 1 | related to salt tolerance |
| *Am022295* | *AT2G45510* | *CYP704A2* | cytochrome P450, family 704, subfamily A, polypeptide 2 | related to salt tolerance |
| *Am023566* | *AT5G36110* | *CYP716A1* | cytochrome P450, family 716, subfamily A, polypeptide 1 | related to salt tolerance |
| *Am024332* | *AT4G31940* | *CYP82C4* | cytochrome P450, family 82, subfamily C, polypeptide 4 | related to salt tolerance |
| *Am030596* | *AT4G31940* | *CYP82C4* | cytochrome P450, family 82, subfamily C, polypeptide 4 | related to salt tolerance |
| *Am030754* | *AT2G46660* | *CYP78A6* | cytochrome P450, family 78, subfamily A, polypeptide 6 | related to salt tolerance |
| *Am029607* | *AT5G51440* | *MFG13_15* | HSP20-like chaperones superfamily protein | related to salt tolerance |
| *Am009922* | *AT2G46150* | *T3F17.20* | Late embryogenesis abundant (LEA) hydroxyproline-rich glycoprotein family | related to salt tolerance |
| *Am009923* | *AT2G46150* | *T3F17.20* | Late embryogenesis abundant (LEA) hydroxyproline-rich glycoprotein family | related to salt tolerance |
| *Am013278* | *AT2G35980* | *YLS9* | Late embryogenesis abundant (LEA) hydroxyproline-rich glycoprotein family | related to salt tolerance |
| *Am028735* | *AT2G46150* | *T3F17.20* | Late embryogenesis abundant (LEA) hydroxyproline-rich glycoprotein family | related to salt tolerance |
| *Am029771* | *AT2G46150* | *T3F17.20* | Late embryogenesis abundant (LEA) hydroxyproline-rich glycoprotein family | related to salt tolerance |
| *Am017663* | *AT1G35830* | *F10O5_2* | VQ motif-containing protein | related to salt tolerance |
| *Am002070* | *AT4G39720* | *T19P19_110* | VQ motif-containing protein | related to salt tolerance |
| *Am010719* | *AT1G35830* | *F10O5_2* | VQ motif-containing protein | related to salt tolerance |
| *Am017558* | *AT1G27730* | *STZ* | salt tolerance zinc finger | related to salt tolerance |
| *Am033679* | *AT1G27730* | *STZ* | salt tolerance zinc finger | related to salt tolerance |
